# Incidence and prevalence of neurofibromatosis type 1 and 2: a systematic review and meta-analysis

**DOI:** 10.1186/s13023-023-02911-2

**Published:** 2023-09-14

**Authors:** Tin-Suet Joan Lee, Meera Chopra, Raymond H Kim, Patricia C. Parkin, Carolina Barnett-Tapia

**Affiliations:** 1 Faculty of Medicine, University of Toronto, Toronto, ON Canada; 2 Elisabeth Raab Neurofibromatosis Clinic, University Health Network, Toronto, ON Canada; 3 Department of Medicine, University Health Network and University of Toronto, Toronto, ON Canada; 4 Institute of Health Policy, Management and Evaluation. Dalla Lana School of Public Health, University of Toronto, Toronto, ON Canada; 5 Department of Pediatrics, The Hospital for Sick Children, University of Toronto, 200 Elizabeth St, Toronto, ON 5EC-334 Canada

**Keywords:** Neurofibromatosis 1, Neurofibromatosis 2, Incidence rate, Prevalence rate

## Abstract

**Objective:**

To obtain updated estimates of the incidence and prevalence of neurofibromatosis type 1 (NF1) and type 2 (NF2).

**Study design:**

We conducted a systematic search of NF1 and NF2 incidence or prevalence studies, in OVID Medline, OVID Embase, Web of Science, and Cinahl. Studies were appraised with the Joanna Briggs Institute Prevalence Critical Appraisal tool. Pooled incidence and prevalence rates were estimated through random-effects meta-analysis.

**Results:**

From 1,939 abstracts, 20 studies were fully appraised and 12 were included in the final review. Pooled NF1 prevalence was 1 in 3,164 (95%CI: 1 in 2,132-1 in 4,712). This was higher in studies that screened for NF1, compared to identification of NF1 through medical records (1 in 2,020 and 1 in 4,329, respectively). NF1 pooled birth incidence was 1 in 2,662 (95%CI: 1 in 1,968-1 in 3,601). There were only 2 studies on NF2 prevalence, so data were not pooled. Pooled NF2 birth incidence was 1.08 per 50,000 births (95%CI: 1 in 32,829-1 in 65,019).

**Conclusion:**

We present updated estimates of the incidence and prevalence of NF1 and NF2, to help plan for healthcare access and allocation. The prevalence of NF1 from screening studies is higher than from medical record studies, suggesting that the disease may be under recognized. More studies are needed regarding the prevalence of NF2.

**Supplementary Information:**

The online version contains supplementary material available at 10.1186/s13023-023-02911-2.

## Introduction

Neurofibromatosis type 1 (NF1, OMIM: 162,200) and type 2 (NF2, OMIM: 101,000) are autosomal dominant genetic disorders caused by mutations in tumor suppressor genes [1, 2]. These disorders can be inherited or result from *de novo* mutations in germ cells. The genes responsible for NF1 and NF2 are located on chromosome 17 and 22, respectively [3].

The incidence and prevalence of NF1 and NF2 has been studied in several regions. NF1 is commonly accepted to be one of the most common autosomal dominant disorders, with estimated minimum prevalence between 1 and 3,000 to 1 in 4,000 people and incidence of 1 in 2,500 births [4, 5]. The estimated prevalence of NF2 is much lower, approximately 1 in 60,000 [6]. However, there is a wide range of estimates, likely due to variations in the populations studied or how cases were identified. Having more accurate estimates of prevalence and incidence can help with healthcare resource allocation and conducting clinical research, such as planning clinical trials. To fill this gap, we conducted a systematic review and meta-analysis of the existing epidemiological studies with the aim of updating global estimates of incidence and prevalence rates for NF1 and NF2.

## Materials and methods

We conducted a systematic review of all published literature measuring the incidence or prevalence of NF1 and/or NF2. The protocol for the systematic review was registered in PROSPERO in August 2021, and a study protocol was not published. The search strategy was carried out by two authors (TJL and MC) in consultation with a health science librarian; we searched Ovid Medline, Ovid Embase, Cinahl, and Web of Science for all published work on February 19, 2021. Search terms were: “Neurofibromatosis 1”, “Neurofibromatosis 2”, “Von Recklinghausen*”, “Incidence”, “Prevalence” and “Epidemiolog*”; the * denotes truncated terms. We included articles that were peer-reviewed primary studies, reviews, or meta-analyses. Inclusion criteria were abstracts reporting: incidence and/or prevalence of NF1 and/or NF2, cases identified based on fulfilling accepted diagnostic criteria (i.e., the 1988 National Institutes of Health (NIH) criteria for NF1 and the Manchester criteria for NF2), and full-text articles written in English, Spanish, or French [7, 8]. We excluded abstracts reporting studies with only participants without NF, focused on treatment or prevention of NF, not using accepted clinical criteria (e.g. administrative medical codes, patient reports, or membership in patient organization), studies reporting incidence and/or prevalence of NF only in specific disease sub-group, and narrative reviews, case reports, and case studies.

**Fig. 1:**
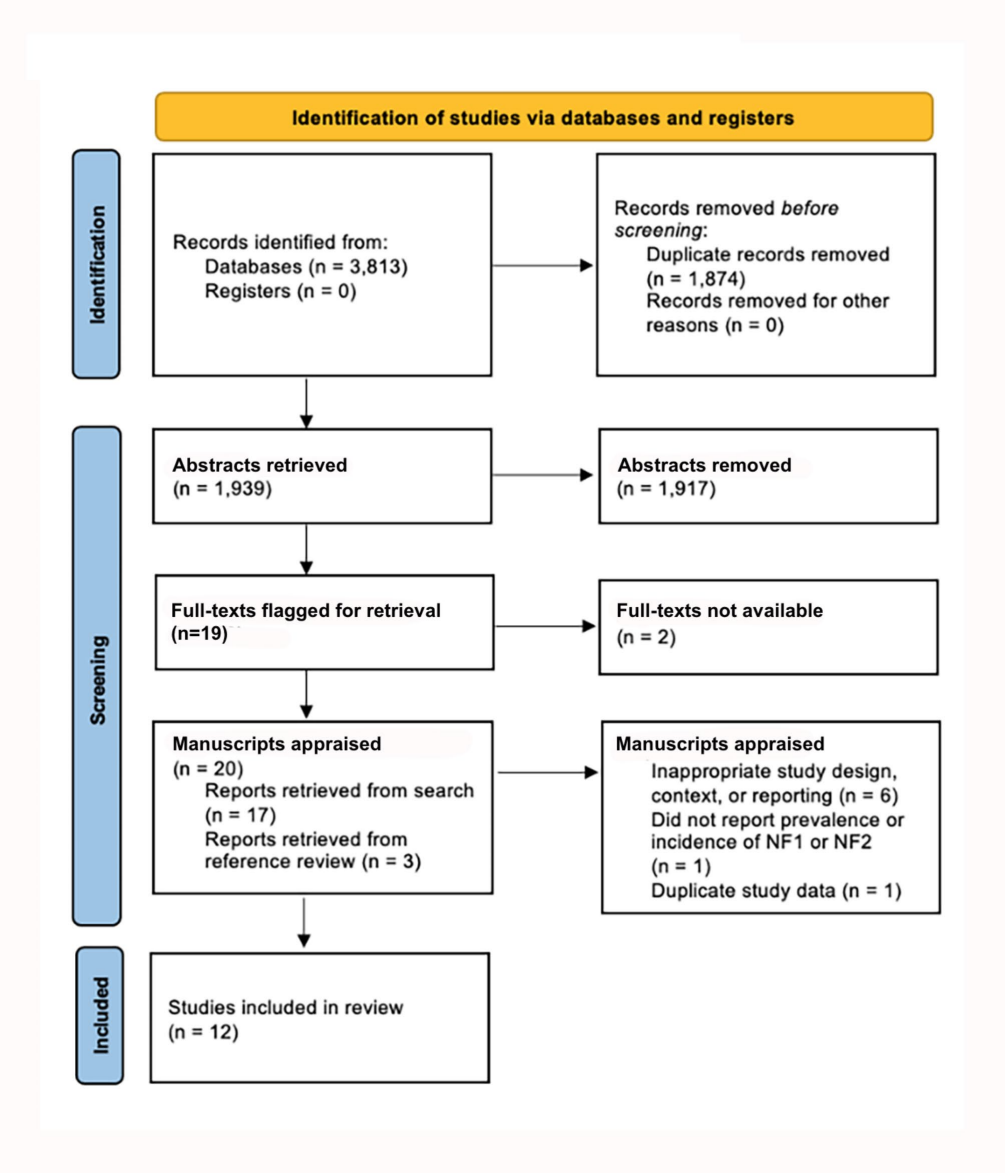
Flow Diagram of Literature Search. PRISMA diagram of the search process, review and final manuscripts included in the review

All abstracts from the primary search were imported into Covidence, an abstract managing site. Duplicate studies were automatically removed by the software, with any remaining duplicates being manually removed by reviewers. Two authors (TJL and MC) independently screened all abstracts to identify studies meeting criteria for full-text review; any conflicts were arbitrated by a third reviewer (CB). Two authors (TJL and MC) then independently screened all full texts to identify studies meeting inclusion criteria. Conflicts were arbitrated by three investigators (PP, RK and CB) who reviewed the selected full-text articles, and used the Joanna Briggs Institute Prevalence Critical Appraisal tool to assess the content and methodological quality of the studies, to determine which studies to include in the review [9]. Studies were excluded due to lack of standardized diagnostic criteria, inappropriate sampling and coverage of the population (i.e. single-centre studies), and insufficient data for appraisal. Details of the search and appraisal process can be seen in Fig. 1; a summary of the studies fully appraised can be seen in Supplementary Table 1. Two investigators (TJL and MC) then independently extracted data from the included studies into a standardized data abstraction form. The data form included the following categories: study design, type of neurofibromatosis, country, population demographics, patient demographics, study setting, sample size, method of measuring sample, response rate, diagnostic technique, number of incident cases, number of prevalent cases, estimate of minimum birth incidence, estimate of minimum prevalence, statistical method, and confounding factors. Additionally, the references from the full-text articles were reviewed and any relevant references that may have been missed in the original search were retrieved for further appraisal.

Because incidence and prevalence may depend on geographical variations, we expected heterogeneity in the studies and estimates. However, given our aim of better understanding the global burden of NF1 and NF2, we conducted meta-analysis if there were > 2 studies for each estimate of interest. For incidence, we only included estimates of birth incidence; if the number of births for the years assessed in the cohort was not in the full-text article, we used publicly available birth statistics for that country and timeframe as denominator. Based on recent recommendations for meta-analysis of single proportions, we used a random-effects model, using generalized linear models with the *metaphor* package for R version 4.2.1 [10, 11]. In cases of multiple studies with a clear difference in ascertainment, for example screening in the general population versus diagnosed cases through health care records, we performed the analyses stratified by group. However, we also performed combined analyses to obtain a global estimate. We assessed heterogeneity using the I [2] statistic that reflects between-study variance and is not affected by sample size [12]. We also assessed publication bias through funnel plots and Egger’s test to test plot asymmetry [10, 11]. The level of statistical significance was 0.05.

## Results

The initial search identified 3,813 records, of which 1,874 duplicates were removed. After abstract review, 17 full-text articles were retained, and another 3 studies were identified from the references. These 20 studies were fully appraised for methodological quality and risk of bias; 12 articles were included in the final review. Of these, two were exclusively done in NF2, eight exclusively in NF1, and two studies included both. Details of the appraised articles and reasons for exclusion are shown in Supplementary Table 1; Fig. 1 depicts the search process.

### NF1

We included 10 studies, of which 9 assessed minimum prevalence, and 3 reported birth incidence. Eight studies were conducted in Europe (Finland = 3, UK = 2, Germany = 1, Italy = 1 and Sweden = 1), one in Cuba and one in Israel. The population sizes studied ranged from 19,392 to 5,400,000 individuals, and studies were published between 1988 and 2018. Five studies ascertained cases through extensive review of medical health records, and 4 studies screened a pre-defined population for clinical criteria for NF1(Table 1).

**Table 1:** Summary of studies included in the review

Reference	Country	Population	Population Size	Birth Incidence	Prevalence Rate
**NF1 Studies using medical records**					
Huson et al., 1988 [13]	United Kingdom	All residents of South-East Wales, age range of 11 months to 83 years.	668,100	NA	1/4949
Samuelsson et al., 1989 [14]	Sweden	All residents of Gothenburg, Sweden, age range of 20 + years.	337,979	NA	1/4600
Poyhonen et al., 2000 [16]	Finland	All residents of Northern Finland, age range 3 months to 73 years (mean 29 years).	733,037	1/3647	1/4436
Evans et al., 2010 [6]	United Kingdom	All residents of Northwest England.	3,050,409	1/2712	1/4560
Uusitalo et al., 2015 [20]	Finland	All residents of Finland.	5,400,000	1/1871	NA
Kallionpaa et al.,2018 [5]	Finland	All residents of Finland.	5,228,552	NA	1/4088
**NF1 studies using screening**					
Garty et al., 1994 [15]	Israel	Jewish recruits for military service, aged 17 years.	374,440	NA	1/960
Lammert et al., 2005 [17]	Germany	Children aged 6 years.	152,819	NA	1/2996
InGordo et al., 2007 [18]	Italy	Military recruits, men.	34,740	NA	1/5735
Orraca et al., 2014 [19]	Cuba	Children aged 9–11 years.	19,392	NA	1/1141
**NF2 studies using medical records**					
Antinheimo et al., 2000 [21]	Finland	All residents in the catchment area of Helsinki University Central Hospital.	1,713,000	1 in 87,410	NA
Evans, 1992 [8]	United Kingdom	All residents of the Northwest Regional Health Authority catchment area.	4,016,000	1 in 40,562	1 in 216,110
Evans et al., 2010 [6]	United Kingdom	All residents of Northwest England.	3,050,409	1 in 33,209	1 in 56,161
Uusitalo et al., 2015 [20]	Finland	All residents of Finland.	5,400,000	1 in 39,336	NA

There were no NF2 studies using screening

**Fig. 2:**
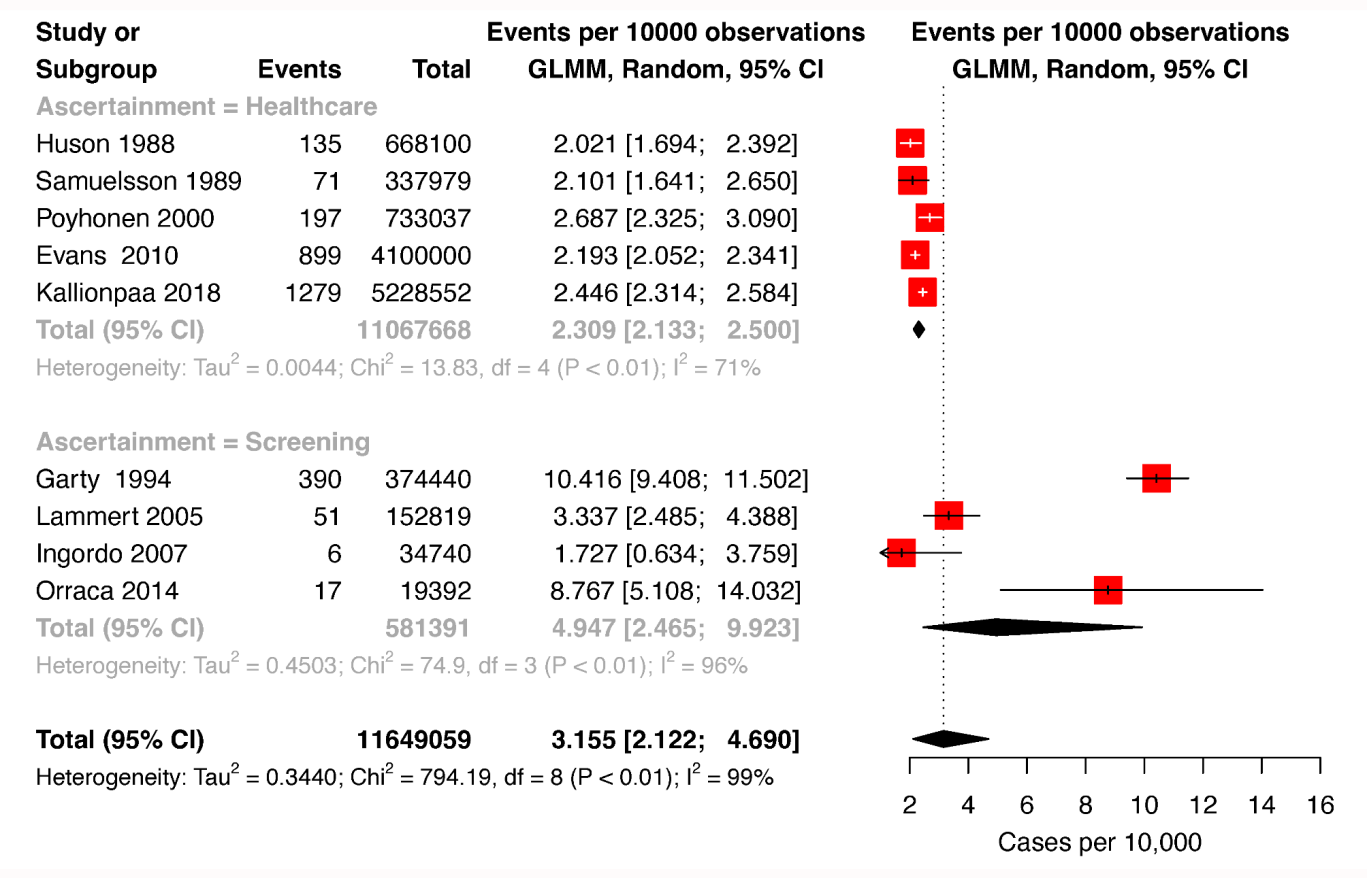
Forest Plots of Random Effects Metanalysis for NF1 prevalence. Prevalence estimates are per 10,000 inhabitants. GLMM: Generalized linear mixed model

We included 9 studies in the meta-analysis of prevalence, with 3,045 cases in a pooled population of 11,649,059 individuals [5, 6, 13–19]. The pooled prevalence estimate for the 9 studies was 3.16 cases per 10,000, (95% CI: 2.12–4.69); this equals to a prevalence of 1 in 3,164; heterogeneity was high with I^2^ = 99%. The sub-group of studies with cases ascertained through medical records had a pooled estimate of 2.31 cases per 10,000 (95% CI: 2.13–2.50), equal to prevalence of 1 in 4,329, with lower heterogeneity (I^2^ = 71%). The subgroup of studies with cases ascertained through screening had a higher estimate of 4.95 cases per 10,000 (95% CI: 2.47–9.92), equivalent to 1 in 2,020; heterogeneity was high in this subgroup (I^2^ = 96%), Fig. 2. Egger’s test p-value was 0.65, indicating a low risk of publication bias.

**Fig. 3:**
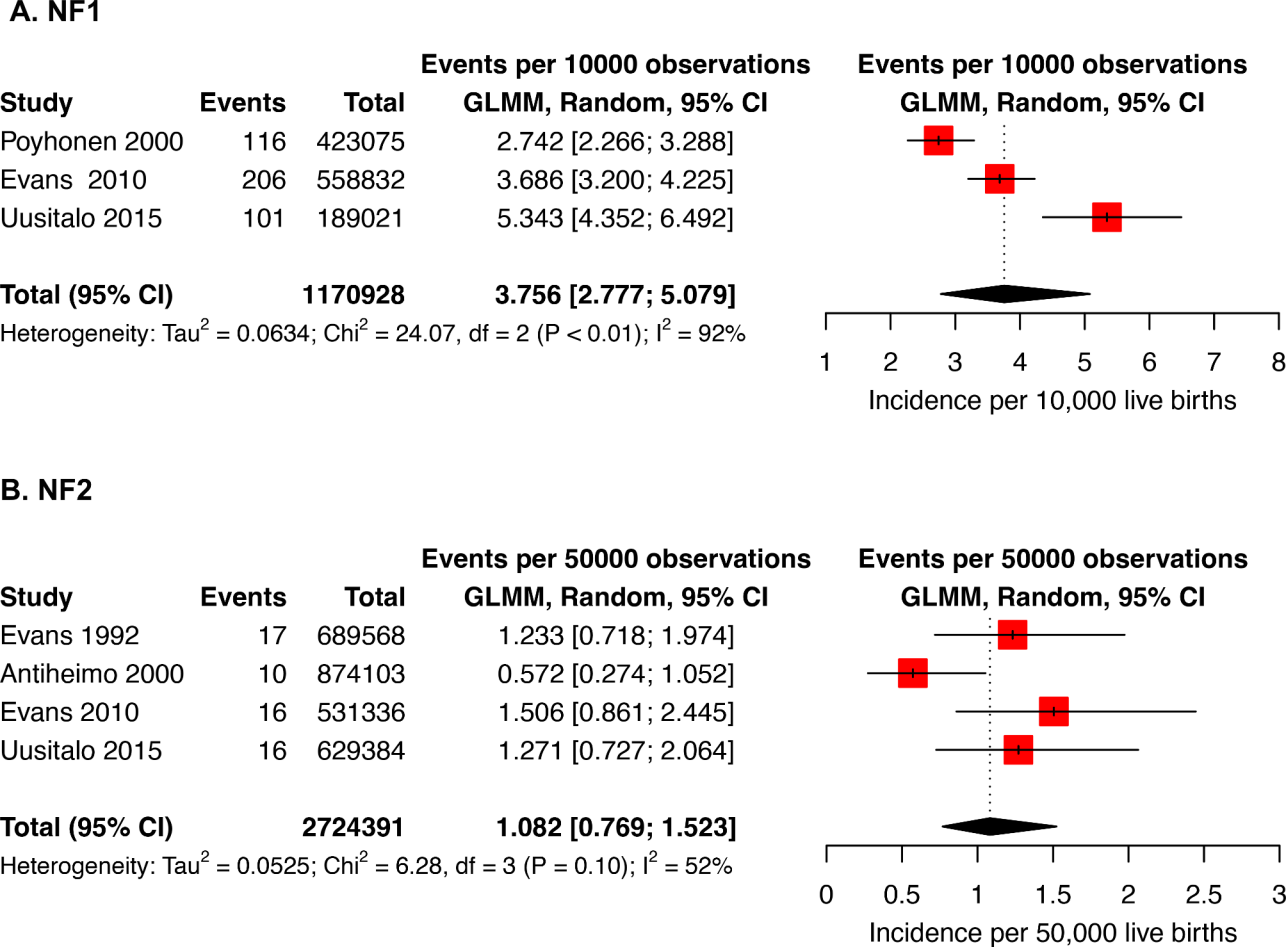
Forest Plot of Random Effects Metanalysis for NF and NF2 birth incidence. Panel **A** depicts estimates for NF1 birth incidence, estimates are per 10,000 live births. Panel **B** depicts estimates for NF2 birth incidence, estimates are per 50,000 live births. GLMM: Generalized linear mixed model

We included 3 studies in the meta-analysis of birth incidence, with 423 cases in 1,170,928 live births [6, 16, 20]. All studies ascertained cases through medical records, and used as denominator the number of live births for the population under study, using age to determine the birth years. The pooled birth incidence estimate was 3.76 per 10,000 live births (95% CI: 2.78–5.08); equivalent to 1 in 2,662 births. There was evidence of heterogeneity with I^2^ = 92% (Fig. 3). Egger’s test p-value was 0.88, indicating a low risk of publication bias.

### NF2

We included 4 studies, of which 2 assessed NF2 prevalence, and all 4 reported live birth incidence. All studies were conducted in Europe (UK = 2 and Finland = 2), and cases were ascertained through extensive medical record review. The populations studied ranged from 1,713,000 and 5,400,000, and studies were published between 1992 and 2015 (Table 1). The 2 studies reporting NF2 prevalence had crude estimates of 1 in 216,110 and 1 in 56,161; we did not pool these estimates [6, 8].

We included all 4 studies reporting NF2 birth incidence in the meta-analysis, for a total of 56 cases in 2,724,391 births [6, 8, 20, 21]. The pooled estimate was 1.08 per 50,000 live births (CI:0.77–1.52), with some heterogeneity (I^2^ = 52%, Fig. 3). Egger’s test p-value was 0.76, indicating a low risk of publication bias.

## Discussion

Our pooled estimate of NF1 prevalence is 1 in 3,190, which is within the range of commonly cited estimates. However, we found that there was marked variability in the prevalence estimates according to ascertainment methods. The estimates from cases identified through medical records reported lower prevalence than estimates from screening studies. This discrepancy between ascertainment methods may indicate that individuals with mild features of NF1 may be missed during routine follow-up and thus patients are not diagnosed; this might reflect lack of provider recognition of the condition or indicate a lack of access to healthcare, which precludes diagnosis. Additionally, given the higher mortality associated with NF1, screening studies—which were done in young individuals— will also result in higher prevalence estimates. Because no epidemiologic study will be able to identify all cases, these estimates are often referred as “minimum incidence/prevalence”. Some studies have used different methods to estimate missing cases and obtain a corrected estimate of incidence or prevalence [5, 13, 16]. However, we only incorporated the observed cases into the analyses, given the variable methods used in the different studies.

NF1 prevalence estimates from health care records were found to be homogenous across studies, whereas NF1 prevalence estimates from screening studies often varied. Homogeneity in the estimates from health care records may reflect the consistent use of diagnostic criteria in these studies, and the pooled estimate likely reflects the prevalence of diagnosed cases. Conversely, high levels of heterogeneity in prevalence rates from screening studies could result from variations in the level of detail of each study’s screening procedure, or demographic characteristics. Age may be the most relevant factor, since screening studies done in children between 9 and 11 and young adults (~ 18 years) showed similar prevalence—around 1:1,000—but a screening study in younger children (6 years old) reported prevalence of ~ 1:3,000 [15, 17, 19]. This is probably due to the fact that some clinical manifestations of NF1 may not be evident in 6-year-olds, thereby precluding making a diagnosis; this can change with a later screening when the most common NF1 manifestations such as café-au-lait macules, skinfold freckling and cutaneous neurofibromas are present in a very high number of individuals [22]. However, one screening study in 18-year-old males assessed before military enrollment in Italy reported lower prevalence (1 in 5,000) [18]. This may reflect geographical differences in prevalence, but very likely reflects selection bias, as individuals with severe features of NF1—such as severe learning disability— are probably not eligible for military service, and thus, would not have been assessed in this study. Therefore, it is possible that true prevalence in the region is higher.

Interestingly, the pooled estimate of prevalence in screening studies (1 in 2,020) is very close to the pooled estimate for birth incidence (1 in 2,662). Arguably, prevalence estimates in children and adolescents though broad screening programs are more reflective of birth incidence, and thus, minimum birth incidence is likely closer to 1 in 2,000. We conducted a sensitivity analysis by pooling estimates of birth incidence with estimates from prevalence screening studies in children and adolescents, resulting in pooled birth incidence estimate of 1 in 2,265 (95%CI: 1 in 1,497-1 in 3,428, supplementary Fig. 1).

The fact that the pooled prevalence (including screening and healthcare record studies) of NF1 is lower than the estimated birth incidence, likely reflects known increased mortality associated with NF1 [5, 23]. Previous studies on mortality in NF1 have shown that mean age of death was 8 to 15 years younger than controls which can certainly be reflected in lower prevalence compared to incidence estimates [24, 25]. Another factor that may affect estimates is publication year, as advances in diagnostic techniques and genetic tools, as well as increased awareness of the condition, may have an impact on the number of reported cases [5]. Additionally, mosaic NF1 can present with a mild phenotype and diagnosis of these patients can be challenging, which can also affect estimates. Given the heterogeneity on the data available in the different studies, we were not able to perform meta-regression to assess the effect of these variables in the estimates.

### NF2

Fewer studies have been conducted to determine NF2 incidence and prevalence rates, therefore, the lack of studies that included prevalence rates precludes an accurate pooled prevalence estimate. Our pooled estimate of birth incidence is 1 in 50,000, with relatively low heterogeneity among studies. This low heterogeneity in birth incidence estimates is likely due to similar populations under study, as the four studies included are from only 2 countries. It is possible that birth incidence may be different in other geographical regions. Additionally, true birth incidence is likely higher than the estimates, as in all studies cases were ascertained through medical records, so they may miss young individuals not yet meeting clinical criteria. As in the case of NF1, new diagnostic criteria have been recently published and future epidemiological studies using these may yield different estimates [26].

### Implications and future directions

Considering the discrepancy in prevalence estimates between health care records and screening studies, conducting wide-ranging NF1 screening may prevent missed cases and ensure that individuals with NF1 receive appropriate care. For example, comprehensive skin assessment could be done by family doctors or pediatricians at around age 10, where most individuals with NF1 will have skin manifestations typical of the disease [27, 28]. Increased physician awareness, especially in primary care, can help improve the accuracy of diagnosis, allowing for earlier treatment and better patient outcomes. For example, given the high prevalence of learning difficulties in children with NF1, early diagnosis and assessment can help access additional learning resources [29].

Our pooled estimates should not be considered to reflect global prevalence or incidence of NF1 and NF2, since most studies included in the analyses were done in Europe, so it is possible that other geographic areas may have different estimates. In particular, Finland and the UK are over-represented in the pooled estimates, as several studies were incorporated from those countries. While there is a risk of duplication in some of the cases identified, the included studies were done in different timeframes and with different geographical coverage (e.g. a single region vs. full country) so we decided to keep all in the analyses. Further research in other world regions is needed to determine global estimates and to assess if there is geographic heterogeneity in incidence and prevalence of NF1 and NF2.

Given the wide discrepancy in NF2 prevalence rates between the two included studies, there is need for further research into the epidemiology of NF2, especially in countries outside of Europe. With the advances and availability of genetic testing, and recognition of mosaic cases of NF2, it is possible that future studies will show a higher prevalence of this condition.

### Strengths and limitations

We performed a comprehensive search with the help of a health science librarian, to make sure that no relevant studies were missed. Three clinicians with experience in NF reviewed the flagged studies for content, following a validated guideline for assessment of prevalence studies. We also only included articles that used validated clinical criteria for diagnosing NF1 and NF2. The main limitation is that most studies were conducted in Europe, and therefore, these estimates may not reflect incidence and prevalence in other regions. Additionally, for NF2, there were very few studies so the estimates should be taken with caution, especially for prevalence, as only two studies with very different estimates were included.

In summary, we present updated estimates of the incidence and prevalence of NF1 and NF2 that can help plan for healthcare access and allocation. Future epidemiologic studies using newly published criteria and in different countries will be needed for more accurate estimates.

### Electronic supplementary material

Below is the link to the electronic supplementary material.

Supplementary Material 1

Supplementary Material 2

Supplementary Material 3

## Data Availability

Data sharing is not applicable to this article as no datasets were generated or analyzed during the current study.

## List of abbreviations

NF1: Neurofibromatosis 1
NF2: Neurofibromatosis 2
