# Supplementary material for: Incidence and prevalence of neurofibromatosis type 1 and 2: a systematic review and meta-analysis
Source: Orphanet J Rare Dis. 2023 Sep 14;18:292. doi: 10.1186/s13023-023-02911-2 (PMC10500831; doi:10.1186/s13023-023-02911-2)

Study	Events	Total	Events per 10000 observations GLMM, Random, 95% CI
Garty 1994	390	374440	10.416 [9.408; 11.502]
Poyhonen 2000	116	423075	2.742 [2.266; 3.288]
Lammert 2005	51	152819	3.337 [2.485; 4.388]
Ingordo 2007	6	34740	1.727 [0.634; 3.759]
Evans 2010	206	558832	3.686 [3.200; 4.225]
Orraca 2014	17	19392	8.767 [5.108; 14.032]
Uusitalo 2015	101	189021	5.343 [4.352; 6.492]
**Total (95% CI)**		**1752319**	**4.414 [2.916; 6.680]**

Heterogeneity: $Tau^2 = 0.2833$; $Chi^2 = 269.07$, df = 6 ($P < 0.01$); $I^2 = 98\%$

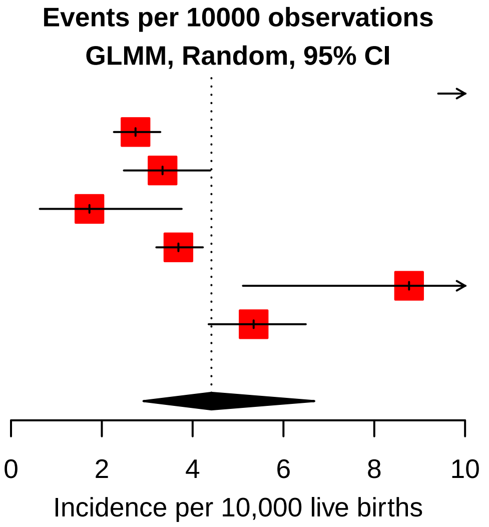

Supplement: Supplementary file 2 — Supplementary Material 2 [file 13023_2023_2911_MOESM2_ESM.pdf]
