# Supplementary material for: Incidence and prevalence of neurofibromatosis type 1 and 2: a systematic review and meta-analysis
Source: Orphanet J Rare Dis. 2023 Sep 14;18:292. doi: 10.1186/s13023-023-02911-2 (PMC10500831; doi:10.1186/s13023-023-02911-2)
Supplement: Supplementary file 3 — Supplementary Material 3 [file 13023_2023_2911_MOESM3_ESM.pdf]

Supplementary Table 1. Full text articles appraised or inclusion

Reference	Country	Population Studied	Include yes/no	Rationale
Sergeyev 1975[1]	Russia	Records form different clinics	No	Cases identified from multiple sources, but no systematically assessed through the population of interest.<br>No diagnostic criteria defined
Huson et al., 1988 [2]	UK	All residents of South-East Wales, age range of 11 months to 83 years.	Yes	Index cases identified through multiple sources. Assessed also family members.<br>Used valid diagnostic criteria.<br>Possibility on under estimating cases, especially in children and people with mild disease who may have not sought medical treatment
Huson et al., 1989 [3]	UK	South-East Wales, South Glamorgan and west Gwent	No	Duplicated data; same population than study by Huson in 1989.
Samuelsson et al., 1989[4]	Sweden	All residents of Gothenburg, Sweden, age range of 20+ years.	Yes	All patients >20 y/o who had sought hospital treatment in the in the city of Gothenburg, Sweden. Large coverage of different specialties and general practitioners. Contacted all doctors in the region.<br>Specific diagnostic criteria used.<br>Limitations: age >20 years but also identified 22 children <20 years; did not provide CIs
Clementi 1990[5]	Italy	North East Italy	No	Only families referred to a single centre for assessment of potential NF1. High risk of missing cases not referred to this clinic or not seeking healthcare.<br>Not NIH diagnostic criteria

Evans 1992[6]	UK	North West England	Yes	Extended ascertainment methods, large coverage through different specialties and clinic. Each individual was assessed individually.<br>NIH criteria used
Garty et al., 1994 [7]	Israel	Jewish recruits for military service, aged 17 years.	Yes	Screening on all military recruits undergoing mandatory health exam. High ascertainment coverage, men and women. Few individuals in the age group would be excluded from assessment.<br>NIH criteria to identify possible cases
Fazii et al. 1998[8]	Italy	Italian men premilitary exam, region of Chiati	No	Study on a large number of military recruits; however, article is a short letter to editor, not enough data on who did screening and who confirmed diagnosis. Did not use NIH criteria
Poyhonen et al., 2000 [9]	Finland	All residents of Northern Finland, age range 3 months to 73 years (mean 29 years).	Yes	Extensive assessment of medical records and doctors in the area, follow up with relatives. NIH criteria used Likely to miss young individuals and those not using the healthcare system (i.e mild disease)
Antinheimo 2002[10]	Finland	100 municipalities around Helsinki in southern Finland.	Yes	Extensive assessment of medical records. Did not assess asymptomatic relatives. Focused on histologically confirmed tumours, may underestimate cases.<br>Used NIH diagnostic criteria

Lammert et al., 2005 [11]	Germany	Children aged 6 years.	Yes	Screening of children age 6. Large coverage of screening.<br>Used NH criteria<br>May miss cases as some children may not yet meet criteria due to age.
Ingordo 2006[12]	Italy	Males 18 y/o, enlisted to Italian Navy, from coastal regions in South Italy.	Yes	Screening of all males assessed for medical exam before being enlisted, high coverage in this population.<br>Predefined NF1 as a diagnosis of interest, screened for NF1 manifestations and referred to specialists to confirm diagnosis using NIH criteria.
McKeever 2008[13]	Ireland	Northern Ireland	No	Only confirmed cases form a single genetic clinic. High risk of missing cases not referred to this clinic or not yet seeking healthcare.<br>Used NIH criteria
Evans et al., 2010 [14]	Northwest England	All residents of Northwest England.	Yes	Large registry from several genetic clinics and also form surveillance from pathology and death certificates.<br>Assessed patients and at-risk relatives<br>Used NIH criteria.<br>May miss very young individuals, and those not seeking healthcare (mild disease)
Rocchetti et al., 2012 [15]	Italy	All residents of Italy.	No	Used NIH criteria.<br>Capture-recapture methods using 2 different sources: a registry and hospital records. Modelling study, no complete ascertainment of cases, high risk of underestimating prevalence.

Orraca et al., 2014 [16]	Cuba	Children aged 9-11 years.	Yes	Screening of children between 9-11 years. Large coverage of population of interest.<br>Used NIH criteria.
Guerra-Jimenez, 2014[17]	Spain	Records from 3 referral hospitals in the region of Cantabria and Las Palmas	No	Limited assessment of cases (only confirmed cases in medical records from 3 hospitals in the region), no follow up with at-risk relatives. High likelihood of underestimating cases. Used available diagnostic criteria
Uusitalo et al., 2015 [18]	Finland	All residents of Finland.	Yes	Extensive search of all medical records in the region. Confirmed each case through clinical review. Used NIH criteria
Kallionpaa 2018[19]	Finland	Medical records of inpatient and outpatient hospital visits in 5 tertiary and 15 secondary referral centers in Finland.	Yes	Used NIH criteria. Prevalence and 95% CIs by 5 year age groups. Ratio of live NF1 patients and population of Finland.<br>Extensive and comprehensive medical record review.
Bata 2019[20]	US	Medical records Olmstead County, Minesota. From 1980-1990	No	Potential cases identified through ICD codes, then extensive chart review. Used NIH criteria. May miss cases without specific ICD codes. Estimated incidence based on newly diagnosed cases, not birth incidence. Unable to calculate birth incidence from paper.

Reference list

1. Sergeyev AS. On the mutation rate of neurofibromatosis. Human Genetics. 1975;28:129-138.

2. Huson SM, Harper PS, Compston DA. Von Recklinghausen neurofibromatosis. A clinical and population study in south-east Wales. Brain : a journal of neurology. 1988;111:1355-1381.

3. Huson SM, Compston DA, Clark P, Harper PS. A genetic study of von Recklinghausen neurofibromatosis in south east Wales. I. Prevalence, fitness, mutation rate, and effect of parental transmission on severity. Journal of Medical Genetics. 1989;26:704-711.

4. Samuelsson B, Samuelsson S. Neurofibromatosis in Gothenburg, Sweden. I. Background, study design and epidemiology. Neurofibromatosis. 1989;2:6-22.

5. Clementi M, Barbujani G, Turolla L, Tenconi R. Neurofibromatosis-1: a maximum likelihood estimation of mutation rate. Human genetics. 1990;84:116-118.

6. Evans DG, Huson SM, Donnai D et al. A genetic study of type 2 neurofibromatosis in the United Kingdom. I. Prevalence, mutation rate, fitness, and confirmation of maternal transmission effect on severity. Journal of medical genetics. 1992;29:841-846.

7. Garty BZ, Laor A, Danon YL. Neurofibromatosis type 1 in Israel: survey of young adults. Journal of Medical Genetics. 1994;31:853-857.

8. Fazii P, Passamonti M, Lappa G, Di Mascio R, Ballone E. Prevalence of neurofibromatosis type 1 among the young adult population of central Italy. Archives of dermatology. 1998;134:1303-1304.

9. Poyhonen M, Kytölä S, Leisti J. Epidemiology of neurofibromatosis type 1 (NF1) in northern Finland. Journal of medical genetics. 2000;37:632-636.

10. Antinheimo J, Sankila R, Carpén O, Pukkala E, Sainio M, Jääskeläinen J. Population-based analysis of sporadic and type 2 neurofibromatosis-associated meningiomas and schwannomas. Neurology. 2000;54:71-76.

11. Lammert M, Friedman JM, Kluwe L, Mautner VF. Prevalence of neurofibromatosis 1 in German children at elementary school enrollment. Archives of dermatology. 2005;141:71-74.

12. Ingordo V, Gentile C, Iannazzone SS, Cusano F, Naldi L. The 'EpiEnlist' project: a dermo-epidemiologic study on a representative sample of young Italian males. Prevalence of selected pigmentary lesions. J Eur Acad Dermatol Venereol. 2007;21:1091-1096.

13. McKeever K, Shepherd CW, Crawford H, Morrison PJ. An epidemiological, clinical and genetic survey of neurofi-

bromatosis type 1 in children under sixteen years of age. The Ulster medical journal. 2008;77:160.

14. Evans DG, Howard E, Giblin C et al. Birth incidence and prevalence of tumor-prone syndromes: Estimates from a UK family genetic register service. American journal of medical genetics Part A. 2010;152A:327-332.

15. Rocchetti I, Taruscio D, Alfò M. Modeling delay in diagnosis of NF: under reportincg, incidence and prevalence estimates. Journal of Applied Statistics

null. 2012;39:711-721.

16. Orraca M, Morejón G, Cabrera N, Menéndez R, Orraca O. Neurofibromatosis 1 prevalence in children aged 9-11 years, Pinar del Río Province, Cuba. MEDICC review. 2014;16:22-26.

17. Guerra-Jiménez G, Camacho PC, Ramos-Macías Á, Angulo CM. Neurofibromatosis type 2 and its head and neck manifestations: literature review and population study in the Community of Cantabria and the province of Las Palmas. Acta Otorrinolaringologica (English Edition). 2014;65:148-156.

18. Uusitalo E, Leppävirta J, Koffert A et al. Incidence and Mortality of Neurofibromatosis: A Total Population Study in Finland. Journal of Investigative Dermatology. 2015;135:904-906.

19. Kallionpää RA, Uusitalo E, Leppävirta J, Pöyhönen M, Peltonen S, Peltonen J. Prevalence of neurofibromatosis type 1 in the Finnish population. Genet Med. 2018;20:1082-1086.

20. Bata BM, Hodge DO, Mohney BG. Neurofibromatosis Type 1: A Population-Based Study. J Pediatr Ophthalmol Strabismus. 2019;56:243-247.
